# *MTNR1B* rs10830963 G Genotype Amplifies Blood Lipid Profile Susceptibility to Poor Circadian Light Hygiene in Arctic Residents

**DOI:** 10.3390/biology15201881

**Published:** 2026-10-18

**Authors:** Denis Gubin, Konstantin Danilenko, Oliver Stefani, Sergey Kolomeichuk, Alexander Markov, Ivan Petrov, Kirill Voronin, Marina Mezhakova, Mikhail Borisenkov, Aislu Shigabaeva, Julianna Petrova, Larisa Alkhimova, Olga Malyugina, Germaine Cornelissen

**Affiliations:** 1 Department of Biology, Tyumen Medical University, Tyumen 625023, Russia; 2 Laboratory for Chronobiology and Chronomedicine, Research Institute of Biomedicine and Biomedical Technologies, Tyumen Medical University, Tyumen 625023, Russia; kvdani@mail.ru (K.D.); h_aislu@mail.ru (A.S.); olya_921@mail.ru (O.M.); 3 Tyumen Cardiology Research Centre, Tomsk National Research Medical Center, Russian Academy of Science, Tyumen 625026, Russia; 4 Institute of Neurosciences and Medicine, Novosibirsk 630117, Russia; 5 Department Engineering and Architecture, Lucerne University of Applied Sciences and Arts, 6048 Horw, Switzerland; oliver.stefani@hslu.ch; 6 Laboratory for Genomics, Proteomics, and Metabolomics, Research Institute of Biomedicine and Biomedical Technologies, Tyumen Medical University, Tyumen 625023, Russia; sergey_kolomeychuk@rambler.ru (S.K.); alexdoktor@inbox.ru (A.M.); lirik92@list.ru (K.V.); chiz.maslova@yandex.ru (M.M.); 7 Laboratory of Genetics, Institute of Biology of the Karelian Science Center of the Russian Academy of Sciences, Petrozavodsk 185910, Russia; 8 Department of Biological & Medical Physics UNESCO, Tyumen Medical University, Tyumen 625023, Russia; petrovtokb@mail.ru (I.P.); pimtmn@mail.ru (J.P.); 9 Department of Molecular Immunology and Biotechnology, Institute of Physiology of the Federal Research Centre Komi Science Centre of the Ural Branch, Russian Academy of Sciences, Syktyvkar 167982, Russia; borisenkov@physiol.komisc.ru; 10 School of Natural Sciences, University of Tyumen, Tyumen 625003, Russia; l.e.alkhim@gmail.com; 11 Department of Integrated Biology and Physiology, University of Minnesota, Minneapolis, MN 55455, USA; corne001@umn.edu

**Keywords:** *MTNR1B*, light exposure, blue light, circadian rhythm, Arctic, blood lipids, triglycerides, seasonal variation, cosinor analysis

## Abstract

Circadian light exposure influences cardiometabolic health, yet the extent to which genetic variation modifies this relationship remains unclear. We investigated whether the MTNR1B rs10830963 genotype was associated with differences in blue-light exposure (BLE) patterns and blood lipid profiles in 63 Arctic residents (35 G-allele carriers) measured around the winter solstice and spring equinox. Participants were grouped by genotype (CC homozygotes vs. G-allele carriers) and contributed week-long actigraphy data analyzed using time-resolved analyses, mixed-effects models, and cosinor modeling. Genotype group differences across seasons were observed in light-hygiene indices, including the normalized amplitude of BLE (NA BLE) and the nocturnal excess index of BLE (NEI). G-allele carriers also exhibited a less favorable lipid profile, with higher morning low-density lipoprotein cholesterol (LDL-C), TG/HDL-C ratio, and Castelli Risk Index I, together with lower HDL-C when the two seasons were considered together. Overall, a larger NA BLE was associated with lower TG, TG/HDL-C ratio, and Castelli Risk Index I, and higher HDL-C, with these associations more evident in G-allele carriers. Time-resolved analyses further showed that associations between morning fasting lipid values and 24 h BLE, assessed across 30 min exposure bins within each season, were rhythmically patterned and more evident in G-allele carriers, with descriptively later acrophases. These findings suggest that MTNR1B rs10830963 may be associated with differences in the relationship between seasonal light exposure and lipid metabolism in Arctic residents.

## Simple Summary

Light exposure is a major environmental cue that shapes circadian biology, with metabolic associations that may depend on genetic background. In this study of Arctic residents exposed to extreme seasonal changes in daylight, carriers of the *MTNR1B* rs10830963 G allele showed distinct 24-h blue-light exposure patterns between winter and spring, particularly during the evening and morning, as captured by novel light-hygiene indices. These patterns were accompanied by differences in blood lipid profiles, including triglycerides, high-density lipoprotein cholesterol (HDL-C), the triglyceride/HDL-C ratio, and Castelli Risk Index I. Notably, associations between the normalized 24 h amplitude of blue-light exposure and lipid outcomes were more evident among G-allele carriers, while time-resolved analyses showed that the strength and timing of blue-light–lipid associations varied according to genotype. These findings suggest that MTNR1B rs10830963 may be associated with differences in how seasonal light exposure relates to lipid metabolism in Arctic populations.

## 1. Introduction

Circadian biology is tightly regulated by environmental light, which acts as the primary synchronizer of internal rhythms to the external day–night cycle [1,2,3]. In high-latitude regions, extreme seasonal fluctuations in photoperiod and light intensity significantly disrupt circadian organization, sleep–wake architecture, and metabolic homeostasis [4,5,6,7,8,9,10,11,12,13]. Consequently, Arctic populations provide a unique natural experiment for examining how varying light exposure, ranging from the profound light deficiency of the winter solstice to the restorative, high-quality hygiene conditions of the spring equinox, modulates physiological regulation [6,8].

Blue-light exposure (BLE) is of particular physiological importance due to its potent activation of melanopsin-containing retinal ganglion cells, which directly entrain the suprachiasmatic nucleus [1,3,8,14,15,16,17]. Beyond its well-characterized roles in phase-shifting and sleep regulation, emerging evidence suggests that light exposure influences systemic cardiometabolic outcomes, including lipid metabolism [8,9,16,18,19,20,21,22,23]. However, the metabolic impact of light likely transcends simple parameters of intensity and timing—it is increasingly understood to be contingent upon individual genetic susceptibility [14,24,25,26,27,28]. However, the extent to which genetic variation modulates the interface between seasonal light environments and lipid homeostasis remains poorly understood. Circadian-related polymorphisms likely underpin inter-individual differences in these physiological responses [3,10,14,24,25,26,27,28]. One notable gene is *MTNR1B*—it encodes melatonin receptor 1B, a G protein-coupled receptor that mediates melatonin signaling and contributes to circadian regulation. The rs10830963 variant in *MTNR1B* is among the most consistently replicated genetic loci associated with fasting glucose and type 2 diabetes risk. Its G-allele is associated with increased *MTNR1B* expression, altered insulin secretion, and impaired glucose tolerance, implicating the locus role in regulating glucose metabolism [14,25,29,30,31,32]. Given its established associations with impaired glucose regulation and circadian rhythmicity, *MTNR1B* may also contribute to metabolic responses to circadian and environmental cues, including light exposure. The *MTNR1B* rs10830963 G-allele is common in European populations, with the proportion of G-allele carriers estimated to be close to 50% [32]. However, its influence on seasonal light–metabolic associations, particularly in the extreme photoperiodic environment of the Arctic, has not been characterized.

In this study, we investigated whether the *MTNR1B* rs10830963 genotype is associated with distinct seasonal BLE patterns and subsequent fasting lipid profiles. We specifically tested whether G-allele carriage modifies the association between circadian light exposure and lipid metabolism in Arctic residents. We hypothesized that G-allele carriers would exhibit distinct seasonal BLE profiles and stronger BLE–lipid associations than CC homozygotes.

## 2. Materials and Methods

This study was approved by the Ethics Committee of Tyumen State Medical University (Protocol No. 101, 13 September 2021) and conducted in accordance with the Declaration of Helsinki. It is a longitudinal observational study involving repeated assessments around the winter solstice and spring equinox. All participants provided written informed consent.

### 2.1. Study Design and Participants

The first phase of the “Light Arctic” study was conducted in Salekhard, Aksarka, and Urengoy, northwestern Siberia, around the winter solstice and spring equinox in 2022–2023. A total of 156 volunteers aged 12–59 years were enrolled. Inclusion criteria were residence at the study site and no recent transmeridian travel, and exclusion criteria were severe or unstable medical conditions, pregnancy or breastfeeding, beta-blocker use, recent acute illness, and age < 3 years. Of the enrolled volunteers, 63 participants met the data-quality criteria and were included in the study. The final sample comprised 40 non-Native and 23 Native participants. Mean age was 40.90 ± 12.65 years, and 77.8% were women. Of the 156 originally enrolled volunteers, participants were eligible for the present analysis only if they completed the required winter solstice and spring equinox assessments and met the predefined actigraphy quality-control criteria. Failure to qualify included voluntary study withdrawal, incomplete seasonal follow-up, protocol violations, relocation from the Arctic study region, newly diagnosed medical conditions meeting exclusion criteria, and insufficient or technically invalid actigraphy data, such as missing actigraphy data exceeding 15% and systematic loss of overnight recordings. Actigraphy recordings were considered inadequate when valid monitoring time was insufficient for the planned 24 h analyses, when prolonged non-wear was present, or when nighttime data were systematically missing. All 63 participants included in the present study had complete, quality-controlled winter and spring data for the primary actigraphy, BLE assessment, genotype, and fasting lipid variables. The final analytical sample was, therefore, determined by longitudinal data completeness and represented the available cohort rather than a sample selected on the basis of an a priori power calculation for testing the present genotype-stratified hypotheses. While the present analysis included participants from the previously established Light Arctic cohort and some actigraphy results overlap with earlier reports, results from specifically examining winter-to-spring differences in 24 h blue-light exposure organization, fasting lipid measures, and their association with the *MTNR1B* rs10830963 genotype are new.

### 2.2. Actigraphy and Light Exposure

Participants wore an ActTrust 2 wrist actigraph (Condor Instruments, São Paulo, Brazil) on the non-dominant wrist for 7 consecutive days and completed sleep diaries. The device recorded motor activity (PIM), wrist temperature, ambient light intensity, and blue-light exposure (BLE, μW ⋅ cm^−2^) in 1 min epochs. Participants were instructed not to cover the device and to press the event marker at bedtime and awakening, and diaries were used to verify timing and exclude artifacts. PIM was used because of its established agreement with polysomnography [33]. Because the device was worn on the wrist, BLE reflects wrist-level light exposure and is an imperfect proxy for corneal light exposure—differences in sensor orientation, clothing, posture, and gaze direction may affect this relationship. Circadian indices derived using ActStudio software Version 1.0.26. included the Midline-Estimating Statistic Of Rhythm (MESOR), 24 h amplitude, acrophase, intra-daily variability (IV), inter-daily stability (IS), relative amplitude (RA), circadian function index (CFI), the mean of the 10 consecutive hours with the highest activity or light exposure (M10) and its onset, and the mean of the 5 consecutive hours with the lowest activity or light exposure (L5) and its onset. Sleep variables included bedtime, wake time, time in bed, total sleep time, sleep efficiency, sleep latency, and wake after sleep onset (WASO), as determined by ActStudio software using standard algorithms [34]. For seasonal analyses, BLE was aggregated into consecutive 30 min epochs and averaged across the 7-day recording.

### 2.3. Blue-Light Exposure Metrics

In addition to standard indices, we calculated three blue-light metrics based on a guideline-relevant daytime/nighttime profile: daylight deficit index for blue light (DDI BLE), nocturnal excess index for blue light (NEI BLE), and normalized amplitude of blue-light exposure (NA BLE). DDI BLE was defined as the integrated deficit below the reference daytime curve between 06:00 and 20:00, and NEI BLE as the integrated excess above the reference curve between 20:00 and 05:00. Reference curves were based on published recommendations for daytime, evening, and nighttime indoor light exposure to best support physiology [35] according to the previously described procedures [36,37,38,39]. NA BLE was calculated as the cosinor-derived 24 h amplitude/MESOR ratio [40]. In this study, “circadian light hygiene” is used as an operational term describing the alignment of objectively measured light exposure with an expected daytime–nighttime pattern. It does not denote a validated behavioral or clinical construct. The construct was quantified using normalized amplitude of blue-light exposure (NA BLE) [40]. DDI BLE and NEI BLE were used for complementary time-resolved analyses.

### 2.4. Genotyping

Genomic DNA was extracted from venous blood using commercial kits according to the manufacturers’ instructions. *MTNR1B* rs10830963 was genotyped by allele-specific real-time PCR on Bio-Rad thermocyclers using fluorescence-based allelic discrimination. Genotype calls were manually reviewed when necessary. The distribution of genotypes was CC = 28 (44.4%), CG = 24 (38.2%), and GG = 11 (17.4%), consistent with Hardy–Weinberg equilibrium (χ^2^ = 2.002, *p* = 0.157). For the primary analyses, participants were grouped according to a dominant genetic model as CC homozygotes and G-allele carriers (CG + GG). This grouping was based on the absence of statistically significant differences between CG and GG participants for the outcomes investigated; however, this does not demonstrate that the two genotype groups are biologically equivalent. It is consistent with the approach used in previous association studies, including our earlier work. Combining CG and GG participants also provides a more stable estimate of the association related to G-allele carriage given the relatively small number of GG participants.

### 2.5. Biochemical Assays

Fasting venous blood was collected between 08:00 and 09:00 after a 12 h fast. Plasma lipid analysis (total cholesterol, TC; high-density lipoprotein cholesterol, HDL-C; low-density lipoprotein cholesterol, LDL-C; triglycerides, TG) was performed by photometry using a Mindray automatic analyzer BS-380 (Mindray, Shenzhen, China). The Castelli Risk Index I (CRI-I) was calculated as TC/HDL-C. Lipid analyses were performed by independent laboratory personnel who had no access to participants’ genotypes or study hypotheses. Statistical analyses were performed using coded datasets, without personally identifiable information.

### 2.6. Data Analysis

The final sample comprised participants from the broader Light Arctic cohort who completed the seasonal protocol and met all data-quality criteria—no separate a priori power calculation was performed for this exploratory genotype-stratified analysis. Models included only the prespecified covariates (age, sex, population, and season) and one outcome at a time. Time-resolved *p*-values were adjusted using the Benjamini–Hochberg procedure at FDR = 0.10. Effect estimates, 95% confidence intervals, and partial η^2^ were reported where applicable. The broader descriptive and genotype-stratified analyses were exploratory and were not subject to formal multiplicity correction; therefore, their results should be interpreted cautiously and require replication.

BMI was included in sensitivity analyses because it may represent either a potential confounder or an intermediate factor linking light exposure to lipid metabolism. The principal models did not include dietary intake, alcohol consumption, smoking, or medication use because these variables were not consistently available for all participants. Sensitivity analyses additionally adjusting for BMI produced associations that were directionally consistent with the primary findings. Because of the limited sample size, models were restricted to a small number of prespecified covariates to avoid overfitting.

Because participants were assessed during both winter and spring, repeated observations were not treated as independent. Longitudinal models included season, genotype, and the genotype × season interaction as fixed effects, with age, sex, and population included as covariates and a participant-specific random intercept to account for within-participant dependence. The genotype × season interaction was used to formally test whether seasonal changes differed between genotype groups.

### 2.7. Statistical Analysis

Normality was assessed using the Shapiro–Wilk test. Between-group comparisons were performed using Student’s *t*-test or one-way ANOVA for normally distributed variables and the Mann–Whitney *U*-test or Kruskal–Wallis test for non-normally distributed variables. Categorical variables were compared using χ^2^ or Fisher’s exact test, as appropriate. Circadian parameters were estimated by cosinor analysis. ActStudio files were used to analyze raw, normalized, and log_10_-transformed actigraphy data by ANOVA and cosinor analysis, as previously described. All tests were two-sided, and *p* < 0.05 was considered to indicate statistical significance. Statistical analyses were performed using STATISTICA 6, SPSS 23.0, and Office Libre Calc (v26.8, The Document Foundation, Berlin, Germany).

Time-resolved BLE–lipid associations were assessed by calculating Pearson correlation coefficients (*r*) between BLE in consecutive 30 min epochs and fasting morning lipid values. The principal aim of the time-resolved analysis was to compare BLE–lipid association profiles between the winter and spring assessments, rather than to test each 30 min interval as an independent hypothesis. The epoch-wise 30 min BLE–lipid analyses and their cosinor summaries were considered exploratory because they involved multiple temporal comparisons based on modest genotype-specific sample sizes. For each lipid outcome and genotype group, the 48 epoch-wise tests were treated as 1 hypothesis family and results were corrected using the Benjamini–Hochberg procedure at FDR = 0.10. Cosinor models were fitted to the epoch-wise *r* profiles to examine rhythmicity of BLE–lipid coupling. Analyses were stratified by the *MTNR1B* rs10830963 genotype. Multivariable models were adjusted for age, sex, population, and season

## 3. Results

### 3.1. MTNR1B rs10830963 Polymorphism Determines Distinct 24 h Light-Exposure Patterns During the Winter Solstice and Spring Equinox in Arctic Residents

Baseline characteristics of the Arctic study participants are presented in Table 1. The CC and G-allele carriers genotype groups were well balanced with respect to age, BMI, sex distribution, and indigeneity, and no significant between-group differences were observed for any of these variables.

**Figure 1:**
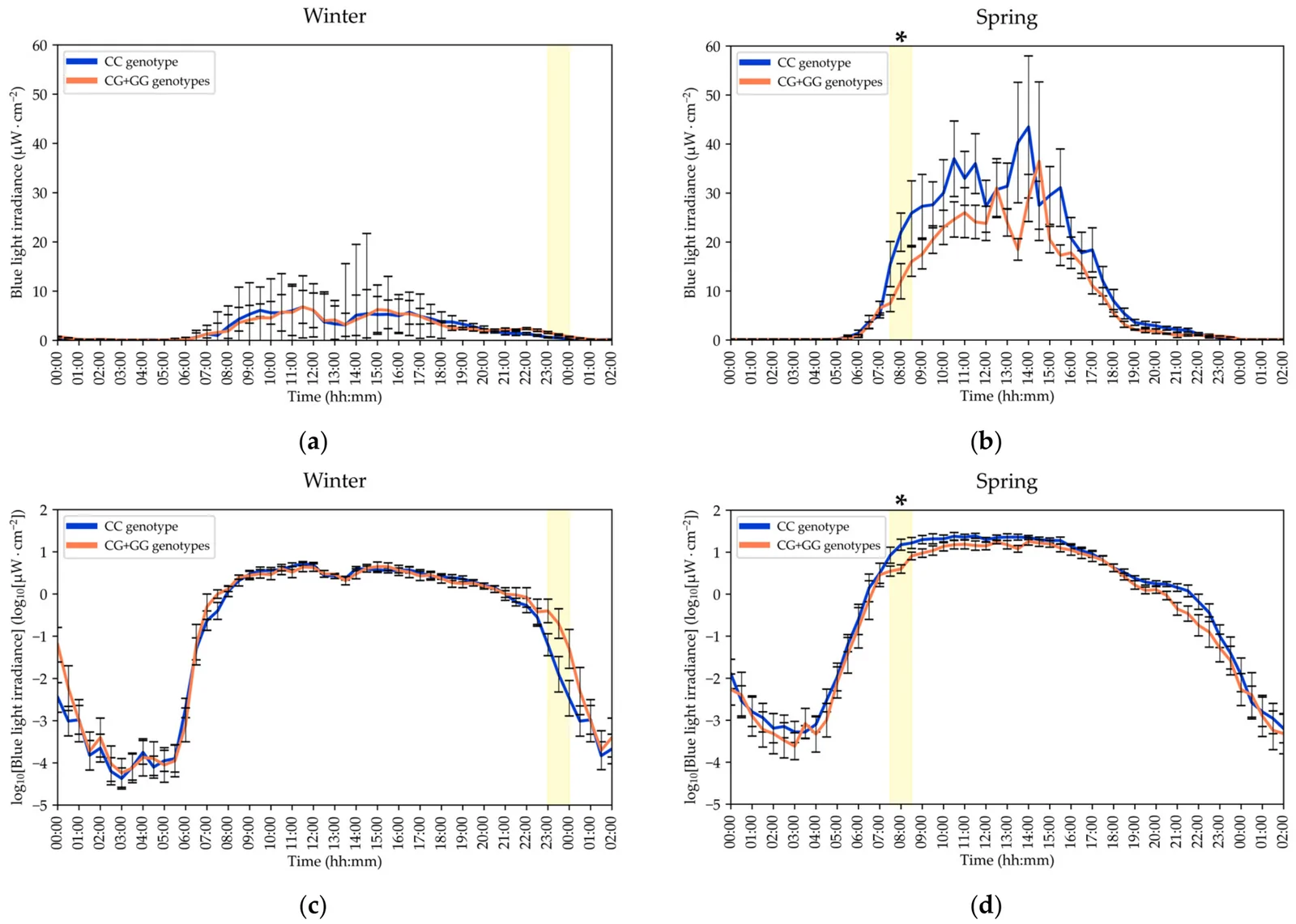
Season-specific 24 h blue-light exposure (BLE) patterns differ by *MTNR1B* rs10830963 genotype. Left panel (winter, (**a**,**c**)): raw BLE irradiance and log_10_-transformed BLE irradiance (log_10_(μW ⋅ cm^−2^)) are shown to emphasize evening low-exposure differences. G-allele carriers (CG + GG) exhibit significantly higher BLE than CC homozygotes between 23:00 and 24:00. Right panel (spring, (**b**,**d**)): G-allele carriers show significantly lower BLE than CC homozygotes between 07:30 and 08:30. Shaded areas indicate time windows with significant group differences by two-sided Mann–Whitney *U*-test. Asterisks (*) mark time windows that remain significant after Benjamini–Hochberg correction for multiple comparisons at false discovery rate (FDR) = 0.10. Curves show median values and standard errors. *MTNR1B*, melatonin receptor 1B gene. The lower segments of CI were removed for plot (**a**).

Distinct 24 h light-exposure patterns, as well as seasonal changes in these patterns, were dependent on the *MTNR1B* rs10830963 genotype. As shown in Figure 1, G-allele carriers exhibited higher evening BLE than CC homozygotes during the winter solstice, particularly between 23:00 and 24:00, whereas lower morning BLE in G-allele carriers was observed during the spring equinox between 07:30 and 08:30. These time-specific differences were identified using Mann–Whitney *U*-tests, and the intervals that remained significant after Benjamini–Hochberg correction are indicated in the figure.

**Figure 2:**
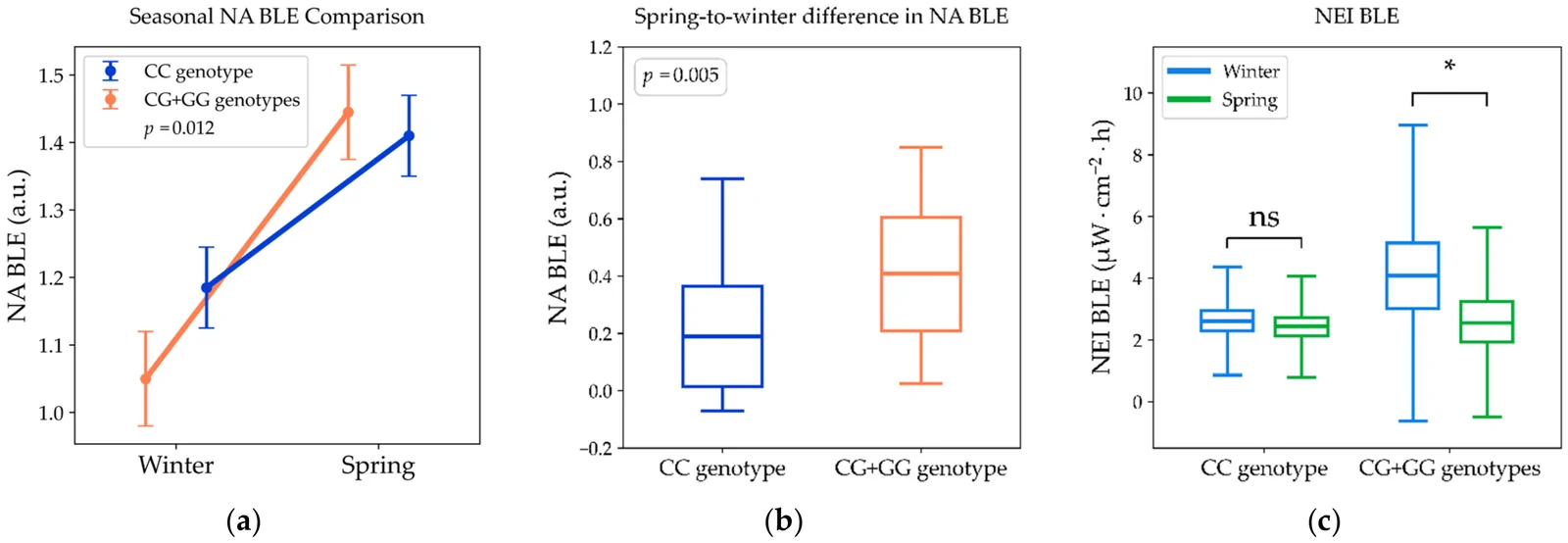
*MTNR1B* rs10830963 genotype and winter-to-spring changes in novel light-hygiene indices. (**a**) NA BLE increased from winter to spring in the full cohort, but the increase was significantly greater in G-allele carriers than in CC homozygotes. The genotype-by-season interaction was significant (two-way ANOVA, *p* = 0.012), and winter NA BLE was lower in G-allele carriers (*p* = 0.048). (**b**) The spring-to-winter difference in NA BLE was greater in G-allele carriers (*p* = 0.005). (**c**) NEI decreased significantly from winter to spring in the within-group analysis in *MTNR1B* rs10830963 G-allele carriers (−1.46; 95% CI −3.01 to −0.09; Wilcoxon *z* = 2.35; *p* = 0.019), whereas no significant seasonal change was observed in CC homozygotes (−0.22, 95% CI −0.78 to 0.35; *p* = 0.191). These within-group seasonal changes do not by themselves establish a significant genotype-by-season interaction. NA BLE, normalized amplitude of blue-light exposure; NEI, nocturnal excess index. Data are presented as mean with 95% CI unless otherwise indicated. * *p* < 0.05; ns – not significant.

In line with these time-specific findings, most summary actigraphy and light-exposure indices did not differ significantly by genotype across seasons (Table 2). The only significant between-genotype difference was observed for the novel circadian light-hygiene index NA BLE, which was lower in G-allele carriers than in CC homozygotes in winter (Figure 2a). The absence of a statistically significant genotype difference in NA BLE during spring does not, by itself, establish that the association was winter-specific—it should be interpreted as an exploratory seasonal pattern. Overall, these findings suggest that *MTNR1B* rs10830963 was associated with differences in the seasonal organization and dynamic range of BLE rather than with broad daily summary measures.

These season-specific differences in 24 h light-exposure patterns were accompanied by *MTNR1B* rs10830963 genotype-dependent changes in the winter-to-spring gradients of the novel light-hygiene indices NEI BLE and NA BLE (Figure 2). NA BLE increased from winter to spring in the entire cohort regardless of genotype (*p* < 0.0001; Figure 2a), but the magnitude of this increase was significantly greater in G-allele carriers than in CC homozygotes (+0.420; 95% CI 0.313–0.527 vs. +0.235; 95% CI 0.145–0.324; *p* = 0.035). Accordingly, the spring-to-winter change in NA BLE was also greater in G-allele carriers (*p* = 0.005; Figure 2b). NEI BLE showed a different pattern, decreasing significantly from winter to spring only in *MTNR1B* rs10830963 G-allele carriers (−1.46; 95% CI −3.01 to −0.09; *z* = 2.35; *p* = 0.019), whereas no significant seasonal change was observed in CC homozygotes (−0.22; 95% CI −0.78 to 0.35; *z* = 1.31; *p* = 0.191; Figure 2c). Together, these findings suggest that *MTNR1B* rs10830963 is associated with genotype-specific seasonal BLE profiles in Arctic residents, characterized by altered evening exposure in winter, reduced morning exposure in spring, and divergent winter-to-spring shifts in novel light-hygiene indices among G-allele carriers.

The normalized amplitude of blue-light exposure (NA BLE) was associated with multiple lipid outcomes in our longitudinal cohort of 63 Arctic residents measured during the winter solstice and spring equinox (Table 3). In the full sample, larger NA BLE predicted lower fasting triglycerides (TG; β = −0.307, 95% CI −0.562 to −0.053, *p* = 0.018), higher HDL-C (β = 0.273, 95% CI 0.037 to 0.509, *p* = 0.023), lower TG/HDL ratio (β = −0.353, 95% CI −0.603 to −0.105, *p* = 0.006), and a lower CRI-I (β = −0.279, 95% CI −0.519 to −0.039, *p* = 0.023). Stratified analyses revealed that these associations were driven primarily by carriers of the *MTNR1B* rs10830963 G-allele (CG + GG): effect sizes were larger and reached significance for TG (β = −0.507, 95% CI −0.942 to −0.071, *p* = 0.024), HDL-C (β = 0.448, 95% CI 0.039 to 0.857, *p* = 0.032), and TG/HDL-C (β = −0.594, 95% CI −1.022 to −0.166, *p* = 0.008). In contrast, corresponding estimates in CC homozygotes were attenuated and non-significant (all *p* > 0.23), indicating that a relationship between NA BLE and lipid metabolism depends on the *MTNR1B* rs10830963 genotype (Table 3). Reported partial η^2^ values indicate small-to-moderate effect sizes for significant associations (partial η^2^ range ≈ 0.046–0.157), and observed power ranged from modest to adequate for the primary findings.

In a complementary analysis, BMI was modeled as an outcome using the same covariates as in the principal lipid models. Higher NA BLE was associated with lower BMI in the full sample (β = −0.341, 95% CI −0.557 to −0.125, *p* = 0.002) and among G-allele carriers (β = −0.518, 95% CI −0.900 to −0.135, *p* = 0.009), whereas the association was not statistically significant among CC homozygotes (β = −0.208, 95% CI −0.516 to 0.101, *p* = 0.183; Supplementary Table S1). These findings were directionally consistent with the principal lipid associations. BMI was not included as a covariate in the lipid models because of its high collinearity with lipid measures and evening light exposure.

Season effects within the NA BLE–lipid mixed-effects models were not statistically significant for any lipid outcome (Supplementary Table S2). Nevertheless, the adjusted spring-to-winter changes consistently tended to be larger among G-allele carriers than among CC homozygotes for all lipid outcomes.

Collectively, these results indicate that the normalized amplitude of blue light exposure is a predictor of TG, HDL-C, TG/HDL-C ratio, and CRI-I primarily in carriers of the *MTNR1B* rs10830963 G-allele, independent of population, sex, age, and season (Table 3; Figure 2).

### 3.2. Circadian Light Hygiene Is Associated with Blood Lipid Profiles in an MTNR1B rs10830963 Genotype-Specific Manner

**Figure 3:**
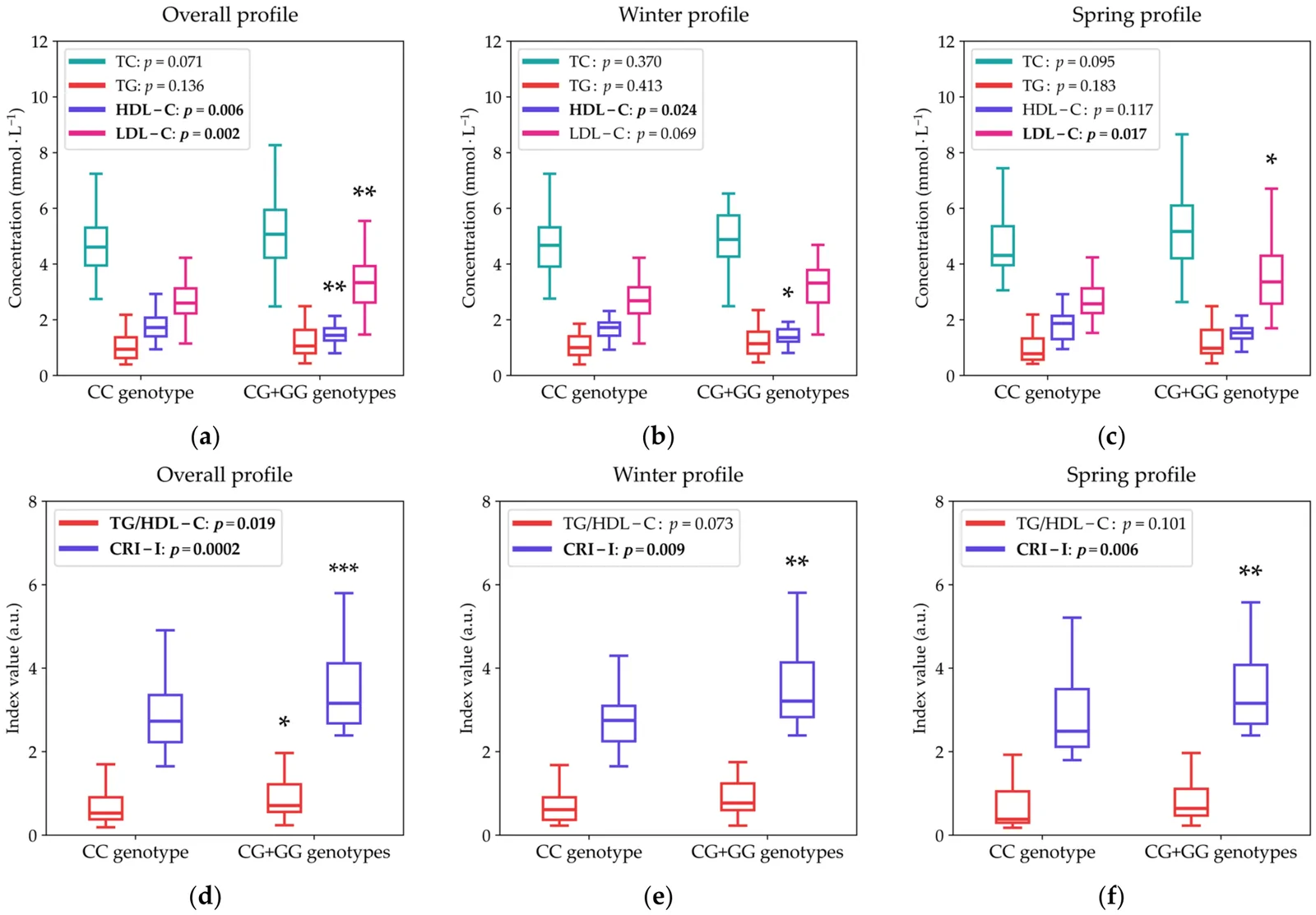
Interplay of *MTNR1B* rs10830963 G-allele carriage with lipid metabolism across seasons. (**a**) TC, TG, HDL-C, LDL-C Overall (winter + spring); (**b**) TC, TG, HDL-C, LDL-C Winter; (**c**) TC, TG, HDL-C, LDL-C Spring; (**d**) TG/HDL-C, CRI-I Overall (winter + spring); (**e**) TG/HDL-C, CRI-I Winter; (**f**) TG/HDL-C, CRI-I Spring. n the two-season longitudinal analysis, *MTNR1B* rs10830963 G-allele carriers exhibit significantly higher morning LDL-C (*p* = 0.002), TG/HDL-C ratio (*p* = 0.019), and CRI-I (*p* = 0.0002), alongside lower HDL-C (*p* = 0.006) compared to non-carriers. Winter: Reduced HDL-C (*p* = 0.024) and borderline elevated LDL-C (*p* = 0.069) contribute to an increased CRI-I (*p* = 0.009) in G-allele carriers during the polar night. Spring: Elevated LDL-C (*p* = 0.017) serves as the primary driver of the significantly higher CRI-I (*p* = 0.006) observed in G-allele carriers during the spring equinox. Note that results are overall consistent between the seasons. Differences in *p*-values around 0.05 are likely due to the modest sampling. * *p* < 0.05, ** *p* < 0.01, and *** *p* < 0.001 (Mann–Whitney *U*-test).

Blood lipid profiles were associated with circadian light hygiene both cross-sectionally within seasons and, more strongly, with the winter-to-spring dynamics of light exposure in an MTNR1B rs10830963 genotype-dependent manner. As shown in Figure 3, G-allele carriers exhibited an adverse lipid profile across the two-season longitudinal analysis, with significantly higher morning LDL-C, TG/HDL-C ratio, and CRI-I, together with lower HDL-C, compared with non-carriers. During the winter solstice, this pattern was driven primarily by reduced HDL-C and a borderline increase in LDL-C, resulting in a higher Castelli Index in G-allele carriers. During the spring equinox, elevated LDL-C emerged as the main contributor to the higher CRI-I observed in G-allele carriers.

Consistent with these group differences, the normalized amplitude of blue-light exposure (NA BLE) was significantly associated with lipid outcomes in the full cohort, but the associations were stronger and more consistent in *MTNR1B* rs10830963 G-allele carriers (Table 3). A larger NA BLE was associated with lower TG (β = −0.307, 95% CI −0.562 to −0.053), lower TG/HDL-C ratio (β = −0.353, 95% CI −0.603 to −0.105), and lower CRI-I (β = −0.279, 95% CI −0.519 to −0.039), as well as higher HDL-C (β = 0.273, 95% CI 0.037–0.509). In stratified analyses, these associations remained significant in G-allele carriers for TG (β = −0.507, 95% CI −0.942 to −0.071), HDL-C (β = 0.448, 95% CI 0.039–0.857), TG/HDL-C (β = −0.594, 95% CI −1.022 to −0.166), and borderline significant for CRI-I (β = −0.386, 95% CI −0.785 to 0.013), whereas no significant associations were observed in CC homozygotes.

Taken together, these findings indicate that circadian light hygiene is linked to lipid metabolism in Arctic residents, and that this relationship is most evident in *MTNR1B* rs10830963 G-allele carriers. Moreover, the stronger associations observed for seasonal changes in light exposure support the notion that not only absolute light exposure but also its temporal organization across winter and spring may contribute to cardiometabolic risk modulation.

### 3.3. Time-Resolved Associations Between Blue-Light Exposure and Morning Fasting Lipid Indices Differ by MTNR1B rs10830963 Genotype

**Figure 4:**
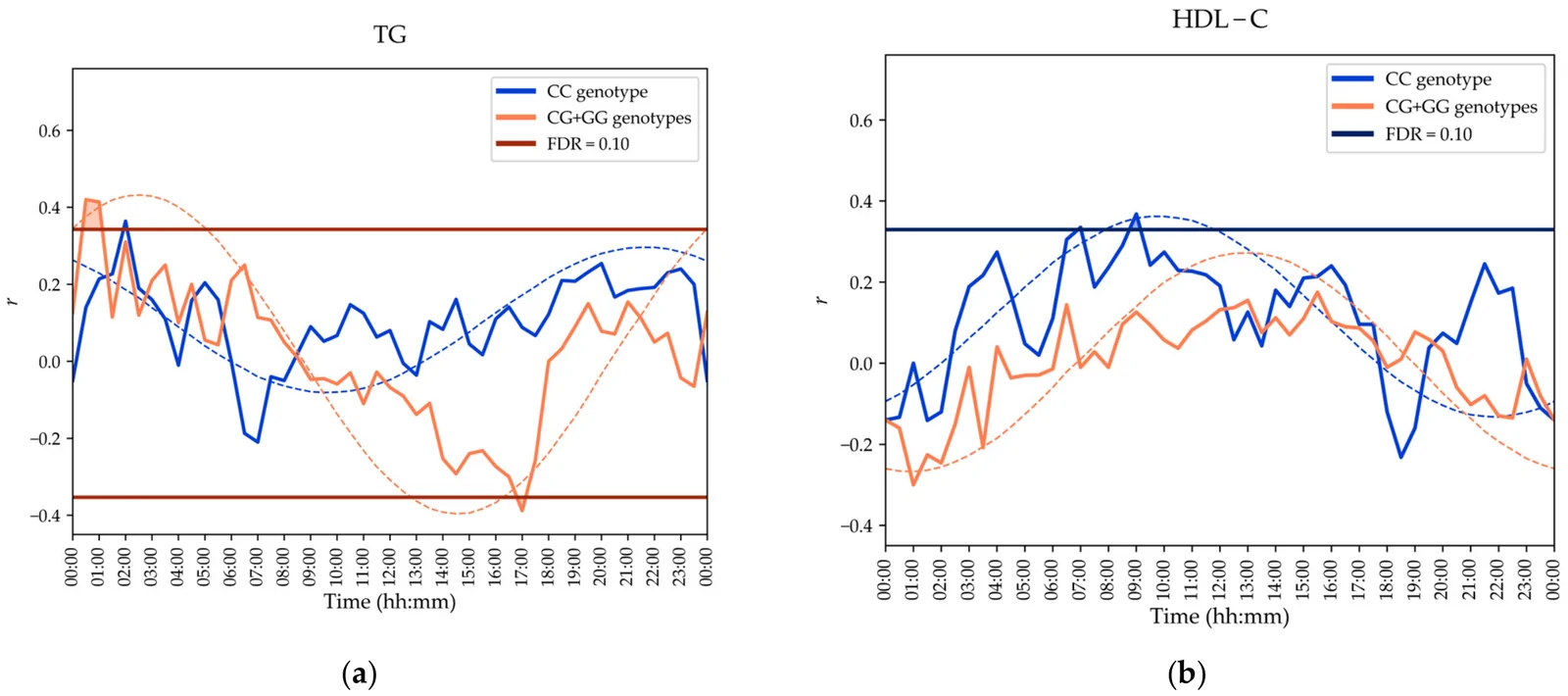

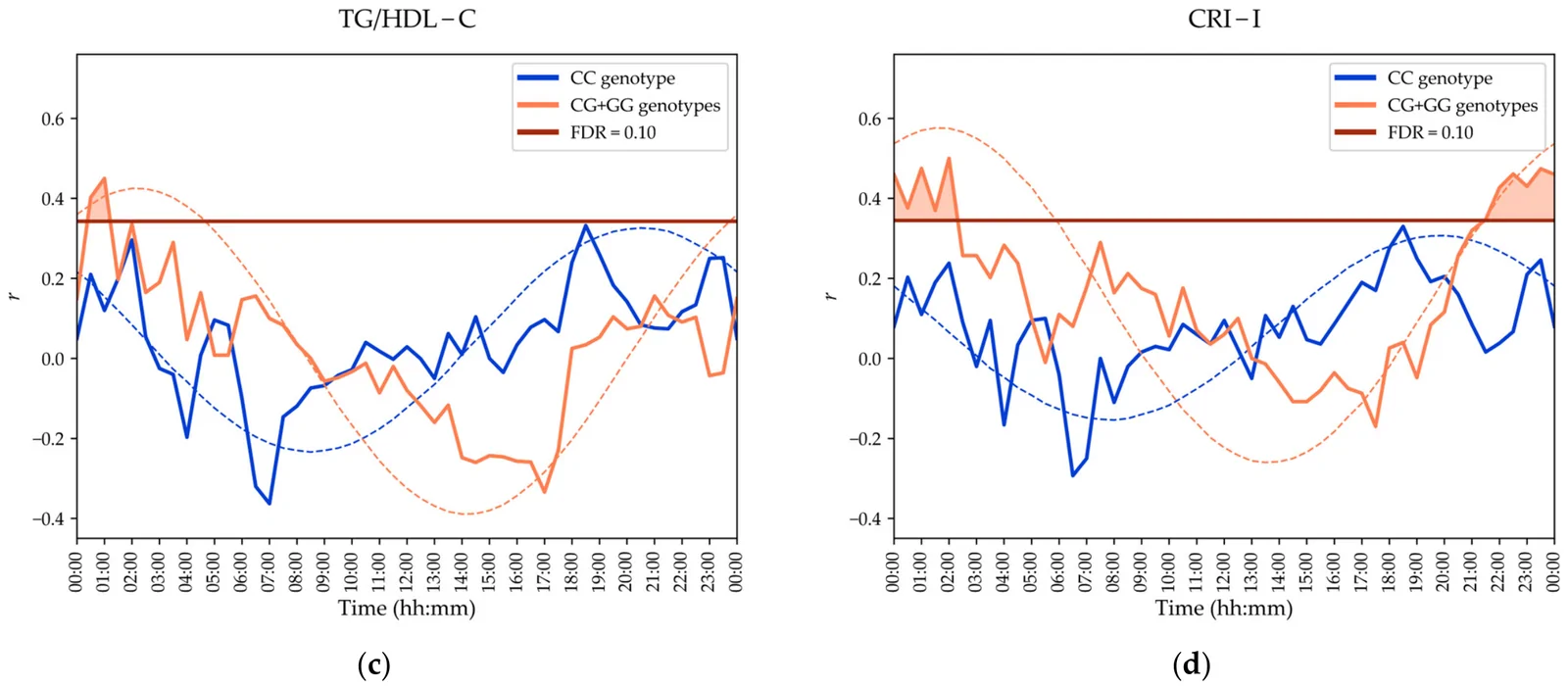
Time-resolved associations between blue-light exposure and morning fasting lipid indices across seasons by *MTNR1B* rs10830963 genotype. Time-resolved Pearson correlation coefficients (*r*) between blue-light exposure (BLE) and morning fasting lipid indices across consecutive 30 min time epochs. Panels show associations with (**a**) triglycerides (TG), (**b**) high-density lipoprotein cholesterol (HDL-C), (**c**) TG/HDL-C ratio, and (**d**) CRI-I. Orange and blue horizontal lines indicate the Benjamini–Hochberg false discovery rate threshold (FDR = 0.10) for G-allele carriers and CC homozygotes, respectively. Time epochs with significant associations after correction are shaded in brown for G-allele carriers and in blue for CC homozygotes. In panel (**a**), a brief significant inverse association between BLE and TG was observed at 17:00–17:30. Orange and blue cosine curves represent the 24 h cosinor fits for G-allele carriers and CC homozygotes, respectively, indicating significant rhythmicity in both genotype groups. The analysis addresses whether morning fasting lipid levels are predicted by BLE across seasonal settings spanning the winter solstice and spring equinox. BLE, blue-light exposure; TG, triglycerides; HDL-C, high-density lipoprotein cholesterol; CRI-I, Castelli Risk Index I; BH, Benjamini–Hochberg correction; FDR, false discovery rate. Horizontal threshold lines denote the FDR-adjusted significance cutoff for each genotype group. Cosinor curves were fitted to the time-resolved correlation profiles and reject the zero-amplitude hypothesis, indicating significant 24 h rhythmicity.

Time-resolved linear regression analyses showed that associations between BLE and morning fasting lipid indices varied across the 24 h cycle and differed by *MTNR1B* rs10830963 genotype (Figure 4). In G-allele carriers, more BLE during evening epochs was associated with higher TG, TG/HDL-C ratio, and CRI-I after Benjamini–Hochberg correction, with the strongest associations concentrated in specific late-day windows. A brief inverse association between BLE and TG was also observed at 17:00–17:30. In contrast, CC homozygotes showed a more limited pattern of significant associations, with the most consistent corrected relationship observed for HDL-C during selected time epochs.

Cosinor modeling confirmed that the time-resolved BLE–lipid associations followed significant 24 h rhythmic profiles in both genotype groups, rejecting the zero-amplitude assumption (Table 4). However, the rhythmic association patterns were stronger and with a later descriptive acrophase in G-allele carriers than in CC homozygotes, consistent with the genotype-dependent differences observed in the seasonal light-exposure analyses and in the NA BLE–lipid associations. Together, these findings indicate that the predictive value of BLE for morning lipid status depends not only on genotype, but also on the timing of exposure across the daily light cycle.

## 4. Discussion

This study shows that the *MTNR1B rs10830963* genotype was associated with distinct seasonal BLE patterns and differences in the relationship between circadian light-exposure patterns and lipid measures in Arctic residents. The principal findings were threefold. First, G-allele carriers exhibited distinct 24 h BLE profiles across seasons, characterized by higher evening exposure in winter and lower morning exposure in spring. Second, the novel light-hygiene indices, particularly NA BLE and NEI, showed different winter-to-spring changes between genotype groups. Third, associations between BLE and fasting lipid profiles were more evident among G-allele carriers, with larger NA BLE associated with a more favorable lipid profile and time-resolved cosinor analyses indicating stronger associations and a later descriptive acrophase of the BLE–lipid profiles in G-allele carriers.

The observed seasonal variations in BLE underscore the challenges posed by the extreme Arctic photoperiod. The transition from the light-deficient winter solstice to the rapidly increasing photoperiod of spring likely impacts circadian entrainment, melatonin dynamics, and metabolic regulation [8,10,14,15,16,17,18,19]. The distinct light-exposure profiles in G-allele carriers suggest that this genotype may influence light-seeking behavior or sensitivity to seasonal environmental constraints. Notably, because broad actigraphy metrics remained stable across genotypes, our data indicate that the *MTNR1B* association is specific to the temporal structure of light exposure, the “circadian hygiene”, rather than global sleep or activity levels. Given that G-allele carriers are known to exhibit delayed melatonin offset [31], our findings suggest that this genetic predisposition may be compounded by altered photic input or impaired light perception [41].

The cardiometabolic relevance of light exposure is supported by experimental and consensus evidence. Recent expert consensus identifies ocular light exposure as a modifiable environmental determinant of health and emphasizes the need for standardized assessment of its timing, intensity, spectral composition, and 24 h distribution [42]. In a controlled laboratory study, exposure to moderate light during sleep increased nighttime heart rate, reduced heart-rate variability, and impaired next-morning insulin sensitivity compared with dim-light sleep [43]. These findings support the biological plausibility of an association between light exposure and metabolic regulation, while also emphasizing that the timing and context of exposure are critical. Our naturalistic Arctic data extend this perspective by examining seasonal 24 h BLE patterns and their associations with fasting lipid profiles rather than experimentally manipulating nocturnal light exposure.

A notable finding of this study was the genotype-specific modulation of novel light-hygiene indices, NA-BLE and NEI. These metrics proved more sensitive than standard daily measures of light exposure, activity, and sleep in capturing the adaptive transition from winter to spring. While NA-BLE increased in both genotype groups, this amplitude change was significantly greater in G-allele carriers. Conversely, NEI decreased solely in G-allele carriers, suggesting a more profound restructuring of their nocturnal light exposure patterns.

Our findings are also consistent with previous evidence that daylight and MTNR1B rs10830963 may jointly relate to metabolic phenotypes. In the prospective PPP-Botnia study, daylight exposure was associated with glycemic traits, and changes in daylight length were related to insulin resistance in rs10830963 G-allele carriers [44]. This earlier work focused on glucose regulation, whereas our study examined seasonal BLE profiles and fasting lipid indices under Arctic photoperiodic conditions. Taken together, the results support the possibility that MTNR1B variation may modify metabolic responses to environmental light, although the specific outcomes and mechanisms may differ across populations and environmental settings. However, because our study was observational, the observed associations cannot establish that *MTNR1B* rs10830963 causes altered light exposure or lipid metabolism. The genotype-specific differences may reflect biological differences in circadian regulation, behavioral responses to seasonal light, or residual confounding—these possibilities require testing in prospective and experimental studies.

This interpretation is strongly supported by the time-resolved analyses, which revealed specific daily windows of BLE association with lipid outcomes, particularly in G-allele carriers. The observed lipid profiles are biologically plausible within the framework of circadian metabolic control. Lipid synthesis, transport, and clearance are demonstrably influenced by disruptions in light timing [8,9,45,46,47,48,49,50]. Our cohort data showed that larger NA-BLE was associated with a more favorable lipid profile (lower triglycerides, TG/HDL-C ratio, and CRI-I, and higher HDL-C), with these beneficial associations being significantly amplified in G-allele carriers. This genotype-specific enhancement suggests that individuals carrying the *MTNR1B* G-allele possess a heightened sensitivity to the metabolic regulatory associations of light timing and its dynamic range.

Time-resolved correlations and cosinor modeling further elucidated the complexity of these interactions. BLE–lipid associations were not uniform throughout the 24 h cycle but instead clustered in specific temporal windows; moreover, cosinor analyses revealed later acrophases and more robust rhythmicity in G-allele carriers. This result suggests that the predictive power of BLE for metabolic status depends not merely on exposure magnitude, but on its precise timing relative to the individual’s internal circadian phase. Such temporal specificity is paramount in Arctic environments, where conventional day–night cues are attenuated or highly unstable. The consistent phase delay observed in BLE–lipid rhythms among G-allele carriers suggests a potential mechanistic role for melatonin signaling in mediating these metabolic outcomes. Furthermore, we identified specific evening windows where excessive BLE was coupled with adverse lipid profiles exclusively in G-allele carriers, while beneficial daylight associations with HDL-C were restricted to the CC genotype, as suggested by the observed association between early morning light and lower HDL-C shown in Figure 4b. These findings underscore the potential for personalized light-hygiene interventions to mitigate metabolic risk.

Several limitations warrant consideration. The modest sample size may limit statistical power for extensive subgroup analyses. The final analysis included 63 participants with complete, quality-controlled seasonal actigraphy, biochemical, and genotype data from the broader Light Arctic cohort. Although observed power for significant associations ranged from 0.49 to 0.80, the modest genotype-specific sample sizes limited power for season-specific, time-resolved, and cosinor analyses and increased the risk of type II error. Multiple testing also increased the risk of type I error, and this issue was addressed by applying Benjamini–Hochberg correction for time-resolved analyses. As this was a longitudinal observational study, causal pathways cannot be definitively established. Finally, while naturalistic light assessment enhances ecological validity, it inevitably introduces behavioral variability. The generalizability of these findings should be considered carefully. Participants were recruited from Arctic populations exposed to exceptionally large seasonal changes in photoperiod and natural light availability. Therefore, the observed genotype-specific light–lipid associations may not apply directly to populations living at lower latitudes or under more stable photoperiodic conditions. Replication in geographically diverse cohorts is needed. Nevertheless, the study’s strengths, including repeated seasonal measurements, genotype stratification, and the application of both summary and high-resolution time-resolved metrics, provide a robust foundation for future investigation into genetic and environmental influences on circadian light exposure and metabolic health.

## 5. Conclusions

This study shows that the *MTNR1B* rs10830963 genotype was associated with seasonal differences in BLE organization and with genotype-specific BLE–lipid associations in Arctic residents. Our findings reveal that the metabolic consequences of BLE are not uniform across the population but are instead contingent upon a complex interplay between temporal light hygiene and genetic background. Specifically, the identified genotype-dependent sensitivity to nocturnal light excess and diurnal light-seeking underscores the role of *MTNR1B* as a critical mediator of circadian metabolic regulation.

These findings suggest that *MTNR1B* rs10830963 may be associated with differences in the relationship between seasonal light-exposure patterns and lipid measures. They do not establish biological mediation or causal metabolic consequences and require confirmation in larger, independently powered cohorts.

## Figures and Tables

**Table 1:** General characteristics ^1^ of Arctic study participants by *MTNR1B* rs10830963 genotype.

Variable	*MTNR1B* CC	*MTNR1B* CG + GG	*p*
Age	41.10 ± 2.09	40.67 ± 2.47	0.892
Body Mass Index (BMI)	24.67 ± 1.04	26.15 ± 1.20	0.355
Sex (Male/Female)	6/22	8/27	0.892
Indigeneity (Native/Non-Natives)	7/21	16/19	0.090

^1^ Data are presented as mean ± s.e. for continuous variables and as n for categorical variables. BMI, body mass index; m/f, male/female. *p*-values were calculated using independent-samples *t*-tests for continuous variables and χ^2^ or Fisher’s exact tests, as appropriate, for categorical variables. Indigeneity was classified as native or non-native. No significant between-genotype differences were observed for age, BMI, sex distribution, or indigeneity.

**Table 2:** Actigraphy and light-exposure metrics ^1^ by *MTNR1B* rs10830963 genotype and season.

Variable	Winter		Spring	
	*MTNR1B* CC	*MTNR1B* CG + GG	*MTNR1B* CC	*MTNR1B* CG + GG
Light Exposure				
LE MESOR	24.7 (19.1; 30.2)	23.0 (16.8; 29.2)	109.1 (74.2; 144.1)	75.3 (58.1; 92.5)
LE Amplitude	27.6 (21.7; 33.5)	23.8 (17.2; 30.3)	150.4 (101.2; 199.6)	106.7 (81.4; 132.0)
LE Acrophase	14:03 (13:35; 14:31)	14:13 (13:35; 14:44)	12:51 (12:33; 13:08)	12:58 (12:34; 13:23)
BLE MESOR	2.78 (2.08; 3.47)	2.50 (1.87; 3.12)	16.64 (11.16; 22.13)	11.69 (8.44; 14.95)
BLE Amplitude	3.18 (2.43; 3.92)	2.57 (1.92; 3.21)	23.68 (15.86; 31.50)	17.05 (12.19; 21.92)
BLE Acrophase	14:04 (13:35; 14:32)	14:07 (13:43; 14:32)	12:46 (12:28; 13:04)	12:53 (12:29; 13:18)
IV BLE	0.722 (0.657; 0.786)	0.783 (0.667; 0.901)	0.948 (0.831; 1.064)	0.902 (0.743; 1.061)
IS BLE	0.432 (0.390; 0.473)	0.412 (0.347; 0.476)	0.373 (0.322; 0.425)	0.411 (0.349; 0.472)
M10 BLE	5.32 (4.09; 6.55)	4.62 (3.47; 5.77)	33.69 (23.07; 44.31)	25.62 (18.45; 32.78)
M10 Onset BLE	08:52 (08:26; 09:18)	08:45 (08:16; 09:14)	07:46 (07:31; 08:02)	07:52 (07:29; 08:16)
L5 BLE	0.131 (0.061; 0.201)	0.145 (0.030; 0.260)	0.084 (0.017; 0.152)	0.083 (0.028; 0.139)
L5 Onset BLE	02:09 (01:39; 02:38)	01:53 (01:12; 02:34)	01:25 (00:39; 02:10)	01:59 (00:37; 03:21)
RA BLE	0.941 (0.915; 0.966)	0.919 (0.851; 0.986)	0.983 (0.973; 0.992)	0.982 (0.970; 0.993)
DDI BLE	349.4 (336.4; 362.4)	357.0 (345.6; 368.4)	203.7 (168.7; 238.7)	212.2 (187.8; 236.5)
NEI BLE	2.48 (1.77; 3.18)	3.78 (1.75; 4.70)	2.45 (1.80; 3.10)	2.55 (1.26; 3.84)
NA BLE	1.17 (1.11; 1.23)	1.03 (0.94; 1.13) *	1.42 (1.35; 1.48)	1.45 (1.40; 1.50)
Activity and Sleep				
MESOR	2473 (2244; 2701)	2686 (2276; 3095)	2581 (2236; 2926)	2828 (2301; 3354)
Amplitude	2038 (1787; 2290)	2074 (1774; 2374)	1758 (1530; 1985)	2018 (1593; 2442)
Acrophase	15:05 (14:30; 15:40)	15:14 (14:52; 15:37)	14:41 (14:09; 15:13)	14:47 (14:16; 15:37)
Bedtime	22:47 (22:13; 23:21)	23:12 (22:37; 23:47)	22:45 (22:19; 23:11)	23:00 (22:19; 23:41)
Wake time	07:41 (07:16; 08:06)	07:34 (07:10; 07:58)	06:59 (06:30; 07:27)	06:52 (06:11; 07:33)
Total Sleep	07:27 (07:08; 07:47)	07:01 (06:33; 07:28)	07:14 (06:51; 07:37)	07:16 (06:40; 07:51)
Sleep Efficiency	88.07 (85.64; 90.50)	86.99 (84.54; 89.45)	88.29 (85.11; 91.48)	88.02 (85.03; 91.02)
WASO	00:57 (00:48; 01:07)	00:55 (00:43; 01:08)	00:54 (00:44; 01:03)	00:52 (00:41; 01:03)
IV	0.878 (0.801; 0.955)	0.872 (0.785; 0.959)	0.863 (0.800; 0.926)	0.861 (0.787; 0.935)
IS	0.524 (0.490; 0.558)	0.562 (0.521; 0.603)	0.541 (0.504; 0.580)	0.541 (0.498; 0.584)
M10	4048 (3592; 4505)	4151 (3503; 4800)	4173 (3692; 4654)	4600 (3717; 5483)
M10 Onset	09:12 (08:43; 09:42)	09:36 (09:08; 10:04)	08:41 (08:13; 09:09)	09:17 (08:43; 09:52)
L5	272.4 (198.9; 345.9)	322.5 (199.1; 446.0)	212.9 (139.4; 286.4)	254.8 (154.2; 355.4)
L5 Onset	02:45 (02:14; 03:17)	02:14 (01:33; 02:54)	01:40 (01:13; 02:07)	01:40 (01:08; 02:12)
RA	0.865 (0.835; 0.896)	0.864 (0.826; 0.901)	0.877 (0.853; 0.902)	0.863 (0.830; 0.896)

^1^ Values are mean (95% confidence interval). Data are shown separately for winter and spring in *MTNR1B* CC homozygotes and G-allele carriers. LE, light exposure, lux; BLE, blue-light exposure, μW ⋅ cm^−2^; MESOR, 24 h rhythm-adjusted mean; amplitude, 24 h amplitude; 24 h acrophase, overall high values recurring each day, as assessed by fitting a 24 h cosine model to the data; M10, mean of the 10 consecutive hours with highest values; L5, mean of the 5 consecutive hours with lowest values; IV, intra-daily variability; IS, inter-daily stability; RA, relative amplitude; DDI, daylight deficit index; NEI, nocturnal excess index. Phase and onset times are reported in hh:mm. DDI and NEI are expressed as μW ⋅ cm^−2^ ⋅ h. The only significant between-genotype difference was observed for the novel circadian light-hygiene index, NA BLE, during winter, with lower values in G-allele carriers than in CC homozygotes (*p* < 0.05), marked *.

**Table 3:** Normalized amplitude of blue-light exposure predicts morning blood lipids only in *MTNR1B* rs10830963 G-allele carriers ^1^.

Outcome	Group	*r*	β [95% CI]	*p*	Partial η^2^
TG	**All**	**–0.202**	**–0.307 [–0.562, –0.053]**	**0.018**	**0.049**
	**CG + GG**	**–0.253**	**–0.507 [–0.942, –0.071]**	**0.024**	**0.116**
	CC	–0.154	–0.131 [–0.488, 0.226]	0.464	0.011
HDL-C	**All**	**0.147**	**0.273 [0.037, 0.509]**	**0.023**	**0.046**
	**CG + GG**	**0.262**	**0.448 [0.039, 0.857]**	**0.032**	**0.104**
	CC	0.072	0.127 [–0.177, 0.432]	0.406	0.014
TG/HDL-C	**All**	**–0.216**	**–0.353 [–0.603, –0.105]**	**0.006**	**0.067**
	**CG + GG**	**–0.328**	**–0.594 [–1.022, –0.166]**	**0.008**	**0.157**
	CC	–0.122	–0.148 [–0.479, 0.183]	0.375	0.015
CRI-I	**All**	**–0.205**	**–0.279 [–0.519, –0.039]**	**0.023**	**0.046**
	**CG + GG**	**–0.204**	**–0.386 [–0.785, 0.013]**	**0.050**	**0.080**
	CC	–0.138	–0.196 [–0.518, 0.127]	0.230	0.028

^1^ Results from linear mixed-effects models using longitudinal data from 63 Arctic residents during the winter solstice and spring equinox. Outcome variables are fasting triglycerides (TG), high-density lipoprotein cholesterol (HDL-C), TG/HDL-C ratio, and CRI-I. Predictor: normalized amplitude of blue-light exposure (NA BLE). Genotype groups: All (full sample), CG + GG (G-allele carriers), and CC (non-carriers). Models adjusted for population, sex, age, and season. Reported statistics: Pearson correlation (*r*), standardized regression coefficient (β) with 95% confidence interval, *p*-value, and partial η^2^ (effect size). Significant associations (*p* < 0.05) are shown in bold. Sample size: *n* = 63 total (CC = 28; CG + GG = 35). Units: TG and HDL-C in mmol ⋅ L^−1^. Abbreviations: NA BLE, normalized amplitude of blue-light exposure; *MTNR1B*, melatonin receptor 1B gene; CI, confidence interval.

**Table 4:** Cosinor model parameters for blue-light exposure–lipid associations by *MTNR1B* rs10830963 genotype.

Outcome	MTNR1B Genotype	Acrophase (hh:mm)	*F*-Statistic	Percent Rhythm	*p*
BLE–TG/HDL-C	CG + GG	02:31	47.19	67.7%	<0.001
	CC	20:30	21.97	49.4%	<0.001
BLE–TG	CG + GG	02:32	42.31	65.3%	<0.001
	CC	21:38	12.27	35.3%	<0.001
BLE–CRI-I	CG + GG	01:38	40.56	64.3%	<0.001
	CC	19:48	17.31	43.5%	<0.001
BLE–HDL-C	CG + GG	12:55	52.21	69.9%	<0.001
	CC	09:51	12.66	36.0%	<0.001

Cosinor model parameters are shown for associations between blue-light exposure (BLE) and morning fasting triglycerides (TG), HDL-C, TG/HDL-C ratio, and CRI-I, stratified by the *MTNR1B* rs10830963 genotype (CC and G-allele carriers, CG + GG). Higher model fit (percent rhythm, equivalent to *R*^2^) and later acrophases indicate stronger and with a later descriptive acrophase. BLE–lipid associations in G-allele carriers. Abbreviations: BLE, blue-light exposure; TG, triglycerides; HDL-C, high-density lipoprotein cholesterol; CRI-I, Castelli Risk Index I; *MTNR1B*, melatonin receptor 1B gene.

## Data Availability

The data presented in this study are available upon reasonable request from the corresponding author. The data are not publicly available due to privacy.

## Supplementary Materials

The following supporting information can be downloaded at: https://www.mdpi.com/article/10.3390/biology15201881/s1. Supplementary Table S1: Normalized amplitude of blue-light exposure predicts body mass index (BMI) in MTNR1B rs10830963 G-allele carriers. Supplementary Table S2: Season effects (spring vs. winter) in linear mixed-effects models assessing associations between NA BLE and morning blood lipids, corrected for population, age, and sex.

## Abbreviations

The following abbreviations and terms are used in this manuscript:

List of abbreviations	
BLE	Blue-Light Exposure (irradiance in the short-wavelength range, measured by the blue channel of the Condor AcTrust2 device)
NA BLE	Normalized Amplitude of Blue-Light Exposure (ratio of the 24 h amplitude of the fitted cosine curve for BLE data to its MESOR, used to standardize the dynamic range of blue-light exposure relative to its average value)
DDI BLE	Daylight Deficit Index for Blue-Light Exposure
NEI BLE	Nocturnal Excess Index for Blue-Light Exposure
BMI	Body Mass Index
CC	CC genotype of *MTNR1B* rs10830963
CG + GG	G-allele carriers of *MTNR1B* rs10830963
CRI-I	Castelli Risk Index I (the ratio of total cholesterol, TC, to high-density lipoprotein cholesterol, HDL-C)
FDR	False Discovery Rate (a statistical method used to correct for multiple comparisons, with a critical value of 0.1 in the study)
HDL-C	High-Density Lipoprotein Cholesterol
LDL-C	Low-Density Lipoprotein Cholesterol
*MTNR1B*	Melatonin Receptor 1B gene
TG	Triglycerides
L5	Least Active/Exposed 5 h Period (the 5 h of lowest values for physical activity or light exposure)
LE	Light Exposure (general light exposure, measured via actigraphy in lux)
M10	Most Active/Exposed 10 h Period (the 10 h of highest values for physical activity or light exposure)
MESOR	Midline-Estimating Statistic Of Rhythm (a rhythm-adjusted mean)
PIM	Proportional Integrative Mode (a method for estimating motor activity in actigraphy)
WASO	Wake After Sleep Onset (time spent awake after initially falling asleep)
List of terms	
Acrophase	Timing of the peak in a circadian rhythm
Amplitude	Magnitude of variation in a circadian rhythm, peak-to-trough difference (e.g., daily light fluctuation)
Chronotype	Individual’s natural preference for morning or evening activity
Actigraphy	Non-invasive wrist-worn device monitoring movement, sleep, and light exposure for objective data
