# Supplementary material for: MTNR1B rs10830963 G Genotype Amplifies Blood Lipid Profile Susceptibility to Poor Circadian Light Hygiene in Arctic Residents
Source: Biology (Basel). 2026 Oct 18;15(20):1881. doi: 10.3390/biology15201881 (PMC13701362; doi:10.3390/biology15201881)
Supplement: Supplementary file 1 [file biology-15-01881-s001.zip › biology-4601775-supplementary.pdf]

Supplementary Table S1. Normalized amplitude of blue-light exposure predicts body mass index (BMI) in *MTNR1B* rs10830963 G-allele carriers.

Outcome	Group	r	β [95% CI]	p	Partial η^2
**BMI**	**All**	**–0.202**	**–0.307 [–0.562, –0.053]**	**0.018**	**0.049**
	**CG+GG**	**–0.253**	**–0.507 [–0.942, –0.071]**	**0.024**	**0.116**
	CC	–0.154	–0.131 [–0.488, 0.226]	0.464	0.011

Supplementary Table S2. Season effects (spring vs winter) in linear mixed-effects models assessing associations between NA BLE and morning blood lipids, corrected for population, age, and sex.

Outcome	Group	β [95% CI]	p
TG	All	–0.169 [–0.417, 0.080]	0.181
	CG+GG	–0.348 [–0.784, 0.087]	0.114
	CC	0.011 [–0.349, 0.370]	0.953
HDL-C	All	0.119 [–0.113, 0.350]	0.312
	CG+GG	0.270 [–0.139, 0.680]	0.191
	CC	0.050 [–0.256, 0.356]	0.744
TG/HDL-C	All	–0.179 [–0.421, 0.064]	0.148
	CG+GG	–0.367 [–0.795, 0.198]	0.092
	CC	–0.017 [–0.349, 0.315]	0.919
CRI-I	All	–0.135 [–0.370, 0.101]	0.259
	CG+GG	–0.252 [–0.674, 0.170]	0.234
	CC	–0.004 [–0.331, 0.322]	0.979
